# Association of systemic inflammatory biomarkers with depression risk: Results from National Health and Nutrition Examination Survey 2005–2018 analyses

**DOI:** 10.3389/fpsyt.2023.1097196

**Published:** 2023-02-08

**Authors:** Xintong Li, Jiaming Huan, Lin Lin, Yuanlong Hu

**Affiliations:** ^1^Faculty of Education, Beijing Normal University, Beijing, China; ^2^First Clinical Medical College, Shandong University of Traditional Chinese Medicine, Jinan, China; ^3^Innovative Institute of Chinese Medicine and Pharmacy, Shandong University of Traditional Chinese Medicine, Jinan, China

**Keywords:** systemic inflammatory biomarkers, systemic immune-inflammation index, systemic inflammation response index, depression, NHANES

## Abstract

**Background/Aim:**

Depression has become a multiple disease worldwide, and is closely related to the systemic inflammatory response.

**Methods:**

Based on the data of the National Health and Nutrition Examination Survey (NHANES), this study included 2,514 depressive and 26,487 non-depressive adults. The systemic immune-inflammation index (SII) and systemic inflammation response index (SIRI) were used to quantify systemic inflammation. The multivariate logistic regression and inverse probability weighting methods were used to analyze the effect size of SII and SIRI on the risk of depression.

**Results:**

After adjusting for all confounders, the above associations of SII and SIRI with depression risk remained significant (SII, OR = 1.02, 95% CI = 1.01 to 1.02, *p* = 0.001; SIRI, OR = 1.06, 95% CI = 1.01 to 1.10, *p* = 0.016). Each 100-unit increase in SII was associated with a 2% increase in the risk of depression, while each one-unit increase in SIRI was associated with a 6% increase in the risk of depression.

**Conclusion:**

Systemic inflammatory biomarkers (SII and SIRI) significantly affected the risk of depression. SII or SIRI can serve as a biomarker of anti-inflammation treatment for depression.

## 1. Introduction

Depression is the most common severe psychiatric illness. According to the Global Burden of Disease Study statistics, 4.4% of the global population had depression in 2017 (1), and the COVID-19 pandemic is estimated to result in 53.2 million additional cases of major depression globally (2). Globally, depression has become the main cause of disability (3). Severe depression is significantly related to shortened life (4, 5), which main reasons are suicide and increased risk of major medical diseases, including cardiovascular disease, diabetes, autoimmune disease, and stroke. Moreover, patients have poor treatment effects for these diseases, which has caused a huge economic burden on society in terms of productivity loss.

In recent years, comprehensive evidence related to depression and the immune system has gradually accumulated (6). Inflammation and depression promote each other and have a significant impact on health. Aggravation of inflammation is an important feature of many cardiovascular and immune metabolic diseases. Multiple meta-analyses (7–9) showed that there were differences in pro-inflammatory cytokines between patients with severe depression and the control group, including IL-6, TNF-α, IL-1β, and CRP. The clinical samples provided a dose–response relationship, indicating that higher IL-6 and CRP predicted the follow-up progress of depression (10). There are also studies suggesting that the excessive inflammatory reaction caused by COVID-19 infection and the chronic inflammation after the symptoms disappear are related to the decline of patients’ emotional state and cognitive ability (11). There are clinical studies (12–15) proving that anti-inflammatory treatment may have an antidepressant effect, but the potential relationship between inflammatory response and depression is very complex. Due to the heterogeneity of research methods, the results of anti-inflammatory treatment are still limited and controversial (16, 17).

Previous studies confirmed that a high level of the systemic immune-inflammation index (SII) was associated with an increased risk of depression in patients with diabetes mellitus (18), stroke (19), tuberculosis (20), and COVID-19 survivors (21). However, the association of systemic inflammatory response biomarkers with depression risk in the whole non-institutionalized population remains unclear, which limited to generalizable to the general population. Thus, we designed an observational study based on the United States population to analyze the relationship between the biomarkers of systemic inflammatory response and the risk of depression.

## 2. Materials and methods

### 2.1. Study design and data source

We designed an observational cross-sectional study based on the data from the National Health and Nutrition Examination Survey (NHANES). NHANES was a cross-sectional survey with a complex multistage sampling design to survey the health and nutritional status of the United States non-institutionalized population (22). The non-institutionalized adults undergoing depression screening from 7 survey cycles (2005–2006, 2007–2008, 2009–2010, 2011–2012, 2013–2014, 2015–2016, and 2017–2018) were included in this study. The participants with missing data were excluded. A total of 29,001 adults were included in the final analysis. Anonymized data from NHANES is freely available for research use at www.cdc.gov/nchs/nhanes/. The NHANES protocol was approved by the National Center for Health Statistics research ethics review board (Protocol #2005–06, Protocol #2011–17, and Protocol #2018–01) (23).

### 2.2. Exposure measures

The systemic inflammation was assessed by calculating two biomarkers, including the SII and systemic inflammation response index (SIRI). The SII was defined as a product of peripheral platelet count (PLA) and neutrophil (NEUT)-to-lymphocyte (LYM) ratio (24), while the SIRI was defined as a product of neutrophil counts and monocyte (MONO)-to-lymphocyte ratio (25).

### 2.3. Outcome measures

The self-reported Patient Health Questionnaire-9 (PHQ-9) was used as a tool to measure the level of depressive symptoms, with good reliability and validity, as demonstrated by a reported Cronbach’s alpha of 0.89 (26). The participants with a PHQ-9 score greater than or equal to 10 were defined as meeting the criteria for depression (27, 28), with a sensitivity of 88%, a specificity of 88%, and a likelihood ratio of 7.1 for major depression (26). The strong measurement invariance for PHQ-9 was reported in NHANES 2005–2016 (29). The PHQ-9 questions were asked by trained interviewers through the Computer Assisted Personal Interviewing (CAPI) system.

### 2.4. Covariate measures

The variates of sociodemographic characteristics, body measure, lifestyle, treatment information, and comorbid disease burden were included in the analyses as covariates, including age, gender, ethnicity, family income, education level, marital status, smoking status, alcohol use, psychological counseling, anti-depression drug use, body mass index (BMI), and the Charlson comorbidity index (CCI).

Family income was divided into two groups according to 130% of the federal poverty level (FPL) as the cut point. The Charlson comorbidity index was the classical method to assess the overall effect of comorbid condition burden (30), including diabetes mellitus, diabetic retinopathy, kidney failure, kidney stones, heart failure, stroke, chronic obstructive pulmonary disease, asthma, chronic bronchitis, liver disease, rheumatoid arthritis, and cancer (Supplementary Table S1). The information on comorbidities was based on self-reports. The variables describing lifestyle, including smoking status and alcohol use, were divided into three categories: never, former, and current. A history of psychological counseling within the past 1 year was identified by asking “During the past 12 months, that is since display current month of display last year, have you seen or talked to a mental health professional such as a psychologist, psychiatrist, psychiatric nurse or clinical social worker about your health?”

### 2.5. Statistical analysis

Participants with missing covariates data were regarded as completely random missing values and removed from analyses. The logistic regression models were used to assume whether the two biomarkers were significantly associated with an increased risk of depression. In addition, restricted cubic spline regression was performed to explore the potential non-linear dose–response association of the two biomarkers with depression risk.

Multivariate logistic regression and inverse probability weighting (IPW) methods were used to correct for covariates. Firstly, a crude model was fitted without adjustment. Then, three multivariate logistic regression models were fitted to control the covariates progressively. Model 1 adjusted for age (<60 years and ≥ 60 years), gender (female and male), ethnicity (white, black, Mexican, and others), education level (below high school, high school or above), family income (<130% FPL and ≥ 130% FPL), marital status (married and non-married), BMI (< 30 kg/m^2^ and ≥ 30 kg/m^2^), and CCI (0, 1 to 3, and > 3). Model 2 adjusted for smoking status (never, former, and current) and alcohol use (never, former, and current). Model 3 adjusted for all confounders, including age, gender, education level, family income, education level, marital status, smoking status, alcohol use, psychological counseling, anti-depression drug use, BMI, and CCI. The main results in the manuscript were presented using model 3. Besides, known confounding effects were minimized by weighting with inverse propensity scores. Inverse propensity scores were calculated based on all confounders by the ipw R package (version 1.0–11), including age, gender, education level, family income, education level, marital status, smoking status, alcohol use, psychological counseling, anti-depression drug use, BMI, and CCI.

Subgroup analyses were performed for age (<60 years and ≥ 60 years), gender (female and male), ethnicity (white, black, Mexican, and others), family income (<130% FPL and ≥ 130% FPL), education level (below high school, high school or above), marital status (married and non-married), smoking status (never, former, and current), alcohol use (never, former, and current), BMI (<30 kg/m^2^ and ≥ 30 kg/m^2^), psychological counseling (No and Yes), anti-depression drug use (No and Yes), and CCI (0, 1 to 3, >3) subgroups. For the subgroup analyses, the interactions were tested using multiplicative interaction terms. To evaluate the potential bias of the exclusion of cases with missing data, missing data of covariates were imputed using multiple imputations by chained equations, and imputed data were used to re-analyze the effect of two biomarkers on depression risk. Multiple imputation was performed using the mice R package (version 3.14.0). E-values were calculated to evaluate the residual measured and unmeasured confounders using the episensr R package (version 1.1.0) (31). Besides, we re-evaluated the association of SIRI and SII with continuous outcomes (PHQ-9 score), and the association of SII and SIRI as dichotomous variables with depression risk. The SII and SIRI were dichotomized based on the mean value.

All statistical analyses and visualization were performed using the R (version 4.2.0)^1^ and RStudio (version 2022.02.3 Build 492)^2^ software. Statistical significance was assessed at a two-sided value of *p* <0.05.

## 3. Results

### 3.1. Population characteristics

**Figure 1:**
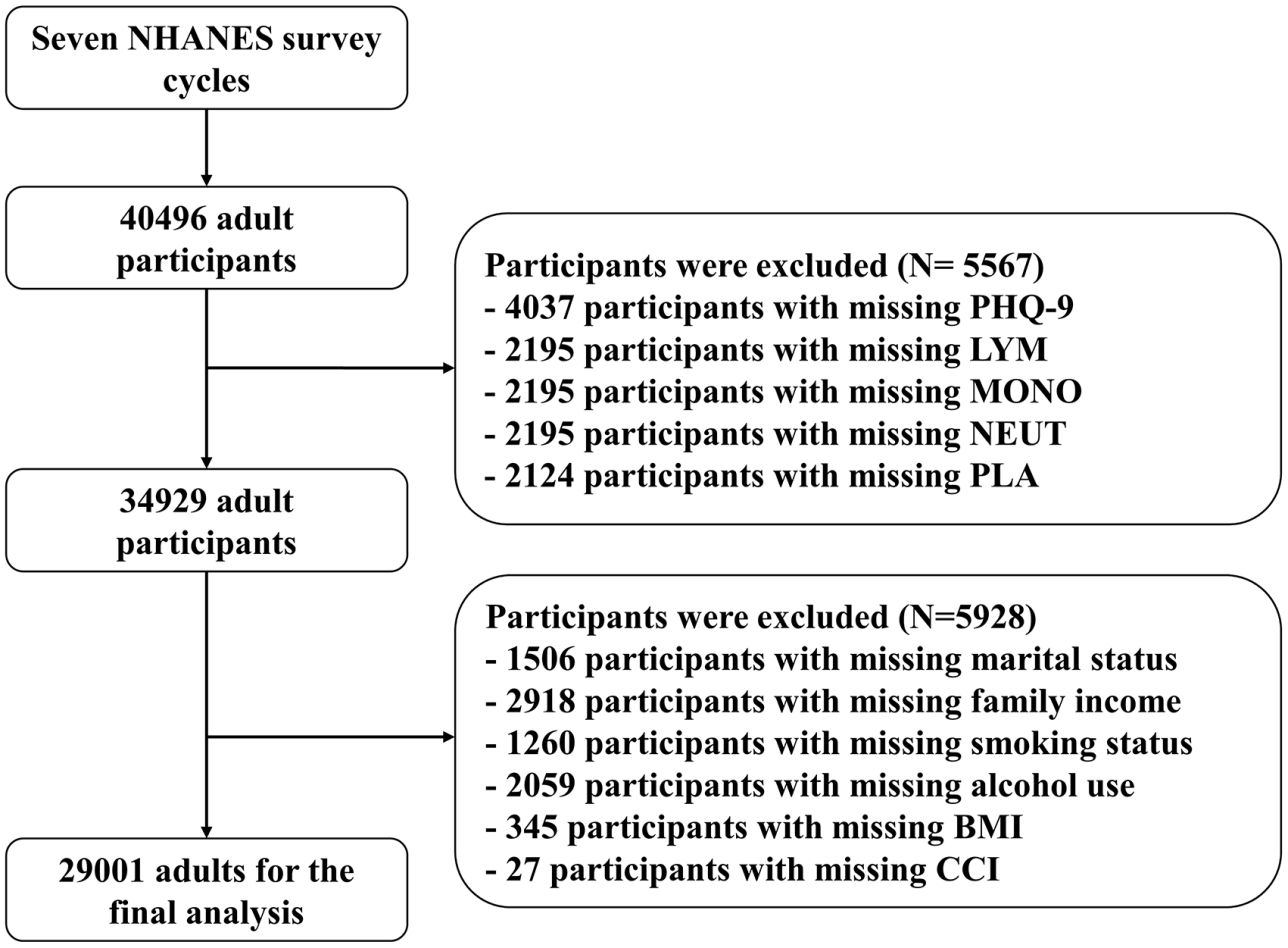
Flowchart of patient inclusion and exclusion.

A total of 34,929 adults were screened for depression using the PHQ-9 in NHANES 2005–2018. After the exclusion of missing covariate values, a total of 29,001 participants were included in the final analyses (Figure 1). Table 1 listed the characteristics of the survey participants. Of these, 2,514 (8.67%) participants with PHQ-9 scores more than or equal to 10 were identified as patients with depression. In total, 14,215 were males (56%) and 14,786 were females (44%). The mean of SIRI was 1.24 (*SD* 0.91) in non-depression and 1.32 (*SD* 0.96) in depression, while the mean of SII was 538 (*SD* 374) in non-depression and 594 (*SD* 415) in depression.

**Table 1:** The characteristics of the survey participants.

Characteristic	Total (*N* = 29,001)	Non-depression (*N* = 26,487)	Depression (*N* = 2,514)	*P*-value
Age, years	49.1 (17.7)	49.2 (17.8)	48.5 (16.0)	0.026^*^
Gender, %				<0.001^***^
Female	14,786 (51.0%)	13,188 (49.8%)	1,598 (63.6%)	
Male	14,215 (49.0%)	13,299 (50.2%)	916 (36.4%)	
Ethnicity, %				0.474
White	12,979 (44.8%)	11,877 (44.8%)	1,102 (43.8%)	
Black	5,943 (20.5%)	5,401 (20.4%)	542 (21.6%)	
Mexican	4,464 (15.4%)	4,088 (15.4%)	376 (15.0%)	
Others	5,615 (19.4%)	5,121 (19.3%)	494 (19.6%)	
Family income, %				<0.001^***^
<130% FPL	8,898 (30.7%)	7,584 (28.6%)	1,314 (52.3%)	
≥130% FPL	20,103 (69.3%)	18,903 (71.4%)	1,200 (47.7%)	
Education level, %				<0.001^***^
Below high school	6,737 (23.2%)	5,874 (22.2%)	863 (34.3%)	
High school or above	22,264 (76.8%)	20,613 (77.8%)	1,651 (65.7%)	
Marital Status, %				<0.001^***^
Married	15,132 (52.2%)	14,240 (53.8%)	892 (35.5%)	
Non-Married	13,869 (47.8%)	12,247 (46.2%)	1,622 (64.5%)	
Smoking status, %				<0.001^***^
Former	15,878 (54.7%)	14,878 (56.2%)	1,000 (39.8%)	
Never	7,100 (24.5%)	6,532 (24.7%)	568 (22.6%)	
Current	6,023 (20.8%)	5,077 (19.2%)	946 (37.6%)	
Alcohol use, %				<0.001^***^
Never	4,004 (13.8%)	3,701 (14.0%)	303 (12.1%)	
Former	4,745 (16.4%)	4,196 (15.8%)	549 (21.8%)	
Current	20,252 (69.8%)	18,590 (70.2%)	1,662 (66.1%)	
BMI, %				<0.001^***^
<30 kg/m^2^	17,835 (61.5%)	16,574 (62.6%)	1,261 (50.2%)	
≥30 kg/m^2^	11,166 (38.5%)	9,913 (37.4%)	1,253 (49.8%)	
CCI, %				<0.001^***^
0	15,795 (54.5%)	14,853 (56.1%)	942 (37.5%)	
1 to 3	11,101 (38.3%)	9,918 (37.4%)	1,183 (47.1%)	
>3	2,105 (7.26%)	1716 (6.48%)	389 (15.5%)	
Psychological counseling				<0.001^***^
No	26,671 (92.0%)	24,834 (93.8%)	1837 (73.1%)	
Yes	2,330 (8.03%)	1,653 (6.24%)	677 (26.9%)	
Anti-depression drug use				<0.001^***^
No	25,871 (89.2%)	24,158 (91.2%)	1713 (68.1%)	
Yes	3,130 (10.8%)	2,329 (8.79%)	801 (31.9%)	
LYM, 1000 cells/ul	2.18 (2.47)	2.17 (2.57)	2.27 (1.09)	<0.001^***^
MONO, 1000 cells/ul	0.56 (0.21)	0.56 (0.21)	0.57 (0.20)	0.007^**^
NEUT, 1000 cells/ul	4.29 (1.82)	4.26 (1.80)	4.68 (1.99)	<0.001^***^
PLA, 1000 cells/ul	248 (66.3)	247 (65.7)	257 (71.8)	<0.001^***^
SIRI	1.24 (0.92)	1.24 (0.91)	1.32 (0.96)	<0.001^***^
SII	543 (378)	538 (374)	594 (415)	<0.001^***^

FPL, federal poverty level; BMI, body mass index; CCI, Charlson comorbidity index; SII, systemic immune-inflammation index; SIRI, systemic inflammation response index; PLA, peripheral platelet count; LYM, lymphocyte count; MONO, monocyte count; NEUT, neutrophil count. *p < 0.05; **p < 0.01; ***p < 0.001.

### 3.2. Association of systemic immune-inflammation index and systemic inflammation response index with depression risk

**Figure 2:**
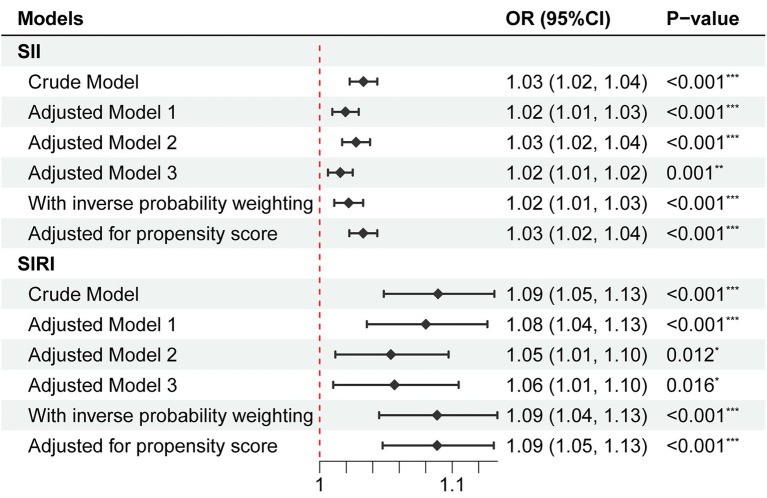
Association of SII and SIRI with depression risk. No adjustment was done in the crude model. Model 1 adjusted for age, gender, ethnicity, education level, family income, education level, marital status, BMI, and CCI. Model 2 adjusted for smoking status and alcohol use. Model 3 adjusted for age, gender, ethnicity, education level, family income, education level, marital status, smoking status, alcohol use, psychological counseling, anti-depression drug use, BMI, and CCI. OR, odds ratio; CI, confidence interval; SII, systemic inflammatory index; SIRI, systemic inflammation response index.

**Figure 3:**
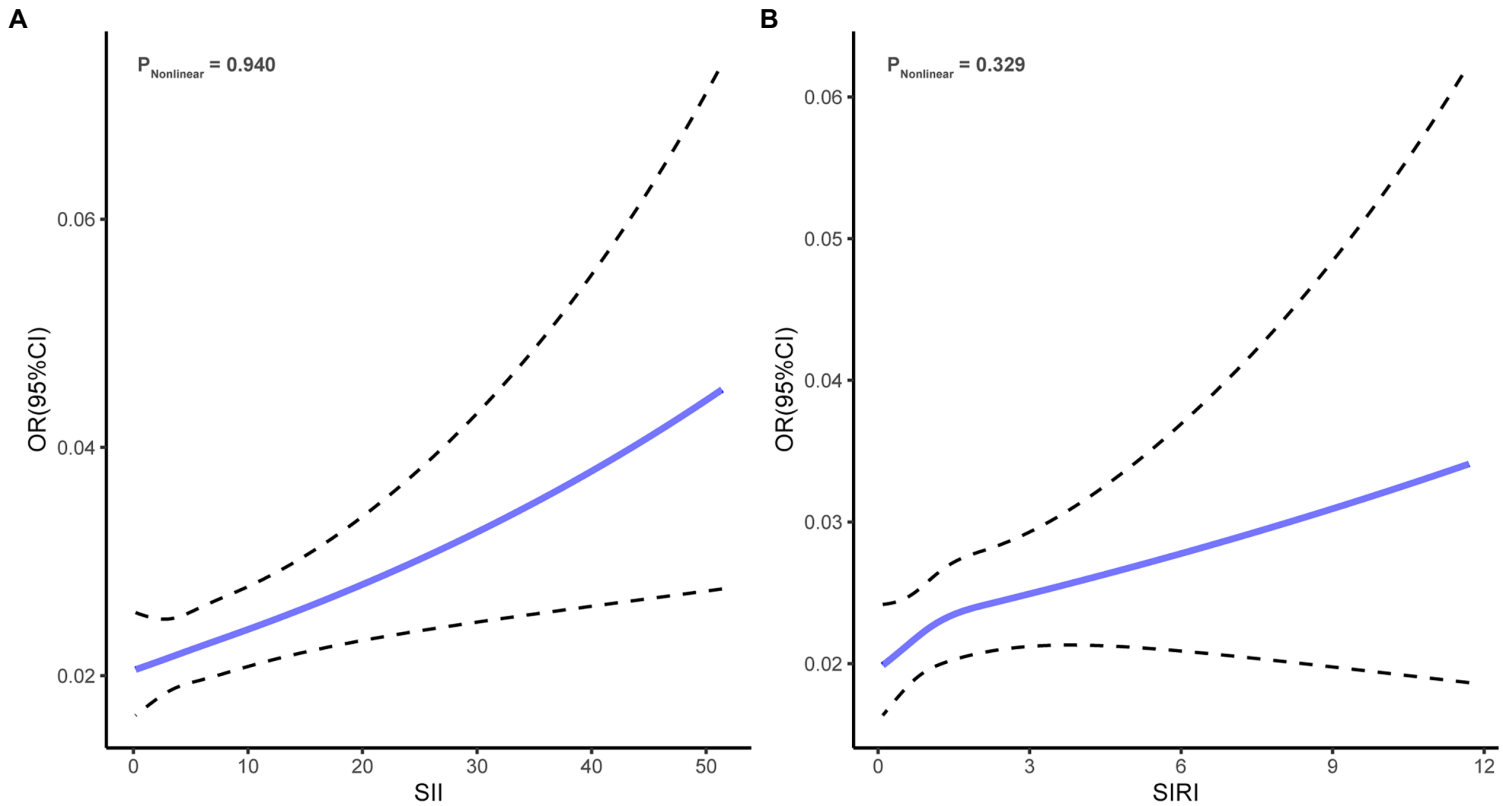
Restricted cubic spline regression curves. **(A)** Association of SII with depression risk. **(B)** Association of SIRI with depression risk. SII, systemic inflammatory index; SIRI, systemic inflammation response index.

As shown in Figure 2, univariate logistic regression analysis for depression risk showed that a high level of SII or SIRI was significantly associated with an increased risk of depression (SII, OR = 1.03, 95% CI = 1.02 to 1.04, *p* < 0.001; SIRI, OR = 1.09, 95% CI = 1.05 to 1.13, *p* < 0.001). After adjusting for all confounders, the above associations remained significant (SII, OR = 1.02, 95% CI = 1.01 to 1.02, *p* = 0.001; SIRI, OR = 1.06, 95% CI = 1.01 to 1.10, *p* = 0.016). The odds ratios indicated that each 100 units increase in SII was associated with a 2% increase in the risk of depression, while each one-unit increase in SIRI was associated with a 6% increase in the risk of depression. In addition, the NEUT, LYM, MONO, and PLA were significantly associated with depression risk (Supplementary Table S2). Restricted cubic spline regression showed no non-linear association of SII (*P*_nonlinear_ = 0.940) and SIRI (*P*_nonlinear_ = 0.329) with depression risk (Figure 3).

We weighted logistic regression models based on inverse propensity scores to adjust for confounders, the association of SII and SIRI with depression risk remained consistent with the results by multivariate logistic regression models (SII, OR = 1.02, 95% CI = 1.01 to 1.03, *p* < 0.001; SIRI, OR = 1.09, 95% CI = 1.04 to 1.13, *p* < 0.001). Besides, we re-fitted the logistic regression models using inverse propensity scores as covariates. The results showed that statistical significance was still maintained (SII, OR = 1.03, 95% CI = 1.02 to 1.04, *p* < 0.001; SIRI, OR = 1.09, 95% CI = 1.05 to 1.13, *p* < 0.001).

### 3.3. Sensitivity and subgroup analyses

**Figure 4:**
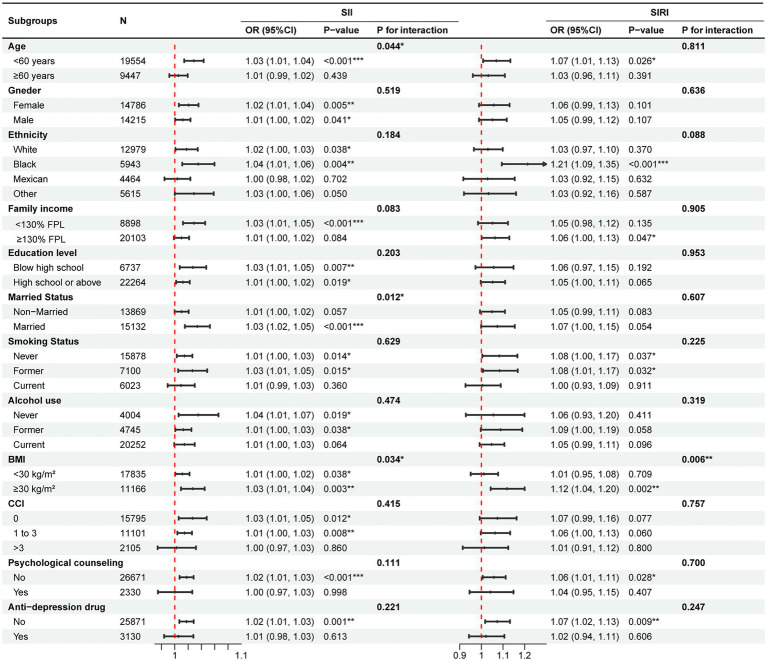
Subgroup analysis for the effect of SII and SIRI on depression risk. Adjusted for age, gender, ethnicity, education level, family income, education level, marital status, smoking status, alcohol use, psychological counseling, anti-depression drug use, BMI, and CCI except for the subgroup variable. FPL, federal poverty level; BMI, body mass index; CCI, Charlson comorbidity index; SII, systemic inflammatory index; SIRI, systemic inflammation response index.

As shown in Figure 4, the results from subgroup analyses showed a consistent direction of effect over all groups. For SII, the interaction tests revealed a non-significant interaction effect across all subgroups except for the age (*P* for interaction = 0.044), marital status (*P* for interaction = 0.012) and BMI (*P* for interaction = 0.034) subgroups. For SIRI, only the interaction effect between SIRI and BMI (*P* for interaction = 0.006) subgroups on depression risk was significant. Notably, a significant interaction was observed in the BMI subgroups for both SII and SIRI, which suggested that systemic inflammatory markers in the obese subgroup (BMI ≥ 30 kg/m^2^) have a higher effect on depression risk than in non-obese subgroups (BMI < 30 kg/m^2^). Besides, for imputed data by multiple imputation, the effect of biomarkers on depression risk remained significant and robust (SII, OR = 1.01, 95%CI = 1.00 to 1.02, *p* = 0.002; SIRI, OR = 1.05, 95% CI = 1.01 to 1.09, *p* = 0.020).

After adjusting for all confounders, participants with high SII or SIRI were associated with a higher risk of depression compared to participants with low SII or SIRI (SII, OR = 1.12, 95% CI = 1.03 to 1.23, *p* = 0.011; SIRI, OR = 1.24, 95% CI = 1.13 to 1.36, *p* < 0.001; Supplementary Table S3). For continuous PHQ-9 score, the results showed that linear associations remained (SII, β = 0.04, 95% CI = 0.02 to 0.05, *p* < 0.001; SIRI, β = 0.11, 95% CI = 0.06 to 0.16, *p* < 0.001; Supplementary Table S4). E-values suggested that the associations of SII and SIRI with depression risk were moderately robust to residual measured and unmeasured confounders (Supplementary Table S5).

## 4. Discussion

We found that an increase in two systemic inflammatory biomarkers was associated with an increased risk of depression after adjustment for confounding factors. It has been shown that depression, SII, and SIRI are, respectively, related to patients’ conditions, lifestyle, treatment information, and disease burden. Therefore, this study adjusted for potential confounders using multivariate logistic regression and inverse probability weighting. After these adjustments, the research model showed a stronger significant correlation. Besides, there was no evidence of nonlinearity dose–response relationships between the biomarkers of systemic inflammatory response and depression risk.

Our study presented evidence that the high level of SII and SIRI were associated with an increased risk of depression. The inflammatory response involves an immune response, blood vessels, and a protective response of molecular mediators. This reaction can be activated by internal and external factors such as microbial infection, atherosclerosis, and ischemia. When the anti-inflammatory medium cannot inhibit the pro-inflammatory reaction, it may develop into a chronic reaction (32, 33). In large clinical studies on inflammatory reactions and the risk of emotional disorders, more discussion on autoimmune diseases such as type 1 diabetes (34) and infectious diseases such as hepatitis (35) increased the risk of depression. Based on large-scale research registered in Denmark (4), emotional disorders after hospitalization due to autoimmune diseases increased by 45%, and the risk of infection after hospitalization increased by 62%. At the same time, studies have shown that there is a two-way correlation between the increased level of peripheral inflammatory factors and depression (10, 36). The increase of CRP and IL-6 in different age groups is related to the increased risk of depression, and the increased level of IL-6 in childhood is related to the occurrence of depression in adulthood (37).

A recent study (38) found that compared with healthy people, the level of nervous system inflammation in patients with active depression increased, which was evaluated by microglial activation. As resting nervous system macrophages, microglia regulate the induction and restriction of neuroinflammatory reactions and play a protective and nutritional role in nerves (39, 40). Many studies have shown that patients with COVID-19 infection have neurodegenerative changes and metabolic abnormalities induced by an inflammatory reaction in the brain (41–43), which lead to autonomic nervous system dysfunction, and are related to depression (44). Some patients with the disappearance of acute COVID-19 infection symptoms still showed elevated inflammatory markers and cognitive impairment (44). In addition, some studies (45, 46) have shown that endothelial cells of the blood–brain barrier can directly or indirectly transmit inflammatory factors, including TNF-α and IL-6, resulting in a two-way communication of inflammation between the peripheral immune system and the central nervous system.

In clinical trials (13, 47), single or combined use of immunosuppressive drugs can produce a better antidepressant effect. Clinical trials in this field mostly focus on non-steroidal anti-inflammatory drugs (NSAIDs) and cytokine inhibitors. A meta-analysis (48) of 36 randomized controlled trials, including 10,000 patients, found that monotherapy plus NSAID, cytokine inhibitor, statins, glucocorticoids, minocycline, and monotherapy all have antidepressant effects, and antidepressants may also benefit from anti-inflammatory drugs (49).

The significance of this study lies in its extension of the relationship between systemic inflammation biomarkers and depression to the general population. It highlights the potential value of measuring systemic inflammatory biomarkers in identifying individuals at risk for depression among the general population, especially those with obesity. The study’s findings also support the idea that anti-inflammatory treatment may be a potential treatment option for depression, as previous research and clinical trials have also suggested. This research provides a better understanding of the relationship between systemic inflammation and depression in the general population and has the potential to inform new diagnostic and treatment options for depression.

There are several limitations to this study. Firstly, this study is limited to the U.S population. Secondly, the use of inverse probability weighting requires a complete data set, so data containing missing values are discarded. However, imputed data by multiple imputation method was re-analyzed to enable robustness. Finally, despite adjustment for many important confounders, the risk of residual measured and unmeasured confounders remains possible, such as the age of first suffering from depression and the history of depression. E-values suggested that a residual measured and unmeasured confounder must be relatively strongly associated with both systemic inflammatory biomarkers and depression risk to completely explain the observed association.

In summary, systemic inflammatory biomarkers (SII and SIRI) significantly affected the risk of depression. SII or SIRI can serve as a biomarker for depression treatment targeting systemic inflammation.

## Data availability statement

Publicly available datasets were analyzed in this study. This data can be found at: https://www.cdc.gov/nchs/nhanes/index.htm.

## Author contributions

All authors contributed to the design, interpretation, and writing of the article and agree to be accountable for all aspects of the work.

## Conflict of interest

The authors declare that the research was conducted in the absence of any commercial or financial relationships that could be construed as a potential conflict of interest.

## Publisher’s note

All claims expressed in this article are solely those of the authors and do not necessarily represent those of their affiliated organizations, or those of the publisher, the editors and the reviewers. Any product that may be evaluated in this article, or claim that may be made by its manufacturer, is not guaranteed or endorsed by the publisher.

## Footnotes

^1^ www.r-project.org

^2^ https://www.rstudio.com/
