# Supplementary material for: Association of systemic inflammatory biomarkers with depression risk: Results from National Health and Nutrition Examination Survey 2005–2018 analyses
Source: Front Psychiatry. 2023 Feb 8;14:1097196. doi: 10.3389/fpsyt.2023.1097196 (PMC9945233; doi:10.3389/fpsyt.2023.1097196)
Supplement: Supplementary file 1 [file Table_1.DOCX]

Supplementary Material

# Supplementary Tables

**Supplementary Table 1. The weights of each comorbidity for CCI.**

| Disease | Weight |
| --- | --- |
| Diabetes mellitus | 1 |
| Diabetic retinopathy | 2 |
| Kidney failure | 2 |
| Kidney stones | 2 |
| Heart failure | 1 |
| Stroke | 1 |
| Chronic obstructive pulmonary disease | 1 |
| Asthma | 1 |
| Chronic bronchitis | 1 |
| Liver disease | 2 |
| Rheumatoid arthritis | 1 |
| Bladder cancer | 2 |
| Bone cancer | 2 |
| Brain cancer | 2 |
| Breast cancer | 2 |
| Cervical cancer | 2 |
| Colon cancer | 2 |
| Esophageal cancer | 2 |
| Gallbladder carcinoma | 2 |
| Kidney cancer | 2 |
| Leukemia | 2 |
| Liver cancer | 2 |
| Lung cancer | 2 |
| Lymphomas | 2 |
| Melanoma | 2 |
| Nervous system cancer | 2 |
| Oral cancer | 2 |
| Ovarian cancer | 2 |
| Pancreatic cancer | 2 |
| Prostatic cancer | 2 |
| Rectal cancer | 2 |
| Skin cancer (non-melanoma) | 2 |
| Other skin cancer | 2 |
| Soft tissue cancer | 2 |
| Stomach cancer | 2 |
| Testicular cancer | 2 |
| Thyroid cancer | 2 |
| Tracheal carcinoma | 2 |
| Endometrial cancer | 2 |
| Other cancer | 2 |

**Supplementary Table 2. T****he associations of NEUT, LYM, MONO, and PLA with depression risk.**

| **Exposure** | **OR (95%CI)** | ***P*-value** |
| --- | --- | --- |
| NEUT, 1000 cells/ul | 1.14 (1.12, 1.16) | <0.001^***^ |
| LYM, 1000 cells/ul | 1.23 (1.16, 1.30) | <0.001^***^ |
| MONO, 1000 cells/ul | 1.36 (1.12, 1.65) | 0.002^**^ |
| PLA, 100, 100000 cells/ul | 1.25 (1.18, 1.33) | <0.001^***^ |

**Notes**: **, <0.01; ***, <0.001.

**Supplementary Table 3. The associations of SII and SIRI as dichotomous variables with depression risk.**

| **Exposure** | **OR (95%CI)** | ***P*-value** |
| --- | --- | --- |
| **SII** |  |  |
| Crude Model | 1.29 (1.18, 1.40) | <0.001^***^ |
| Adjusted Model 1 | 1.17 (1.07, 1.27) | <0.001^***^ |
| Adjusted Model 2 | 1.23 (1.13, 1.34) | <0.001^***^ |
| Adjusted Model 3 | 1.12 (1.03, 1.23) | 0.011^*^ |
| **SIRI** |  |  |
| Crude Model | 1.30 (1.20, 1.41) | <0.001^***^ |
| Adjusted Model 1 | 1.27 (1.16, 1.39) | <0.001^***^ |
| Adjusted Model 2 | 1.20 (1.11, 1.31) | <0.001^***^ |
| Adjusted Model 3 | 1.24 (1.13, 1.36) | <0.001^***^ |

Notes: *, <0.05; ***, <0.001.

**Supplementary Table 4. The associations of SII and SIRI as dichotomous variables with PHQ-9 score.**

| **Exposure** | **β (95%CI)** | ***P*-value** |
| --- | --- | --- |
| **SII** |  |  |
| Crude Model | 0.09 (0.07, 0.10) | <0.001^***^ |
| Adjusted Model 1 | 0.05 (0.04, 0.07) | <0.001^***^ |
| Adjusted Model 2 | 0.08 (0.06, 0.09) | <0.001^***^ |
| Adjusted Model 3 | 0.04 (0.02, 0.05) | <0.001^***^ |
| **SIRI** |  |  |
| Crude Model | 0.19 (0.14, 0.24) | <0.001^***^ |
| Adjusted Model 1 | 0.15 (0.10, 0.20) | <0.001^***^ |
| Adjusted Model 2 | 0.13 (0.08, 0.18) | <0.001^***^ |
| Adjusted Model 3 | 0.11 (0.06, 0.16) | <0.001^***^ |

Notes: ***, <0.001.

**Supplementary Table 5. Robustness to unmeasured confounding (E-values).**

| **Exposure** | **Effect Estimate** | **Confidence Interval Limit** |
| --- | --- | --- |
| SII | 1.53 | 1.39 |
| SIRI | 1.54 | 1.42 |
